# Radiomics combined with transcriptomics to predict response to immunotherapy from patients treated with PD-1/PD-L1 inhibitors for advanced NSCLC

**DOI:** 10.3389/fradi.2023.1168448

**Published:** 2023-05-03

**Authors:** Amine Bouhamama, Benjamin Leporq, Khuram Faraz, Jean-Philippe Foy, Maxime Boussageon, Maurice Pérol, Sandra Ortiz-Cuaran, François Ghiringhelli, Pierre Saintigny, Olivier Beuf, Frank Pilleul

**Affiliations:** ^1^ Department of Radiology, Centre Léon Bérard, Lyon, France; ^2^ Creatis, University Lyon, INSA-Lyon, Université Claude Bernard Lyon 1, CNRS, Inserm, Creatis, UMR 5220, U1206, Lyon, France; ^3^ Department of Oral and Maxillofacial Surgery, Sorbonne Université, Pitié-Salpêtrière Hospital, APHP, Paris, France; ^4^ Department of Medical Oncology, Centre Léon Bérard, Lyon, France; ^5^ CRCL, University Lyon, Claude Bernard Lyon 1 University, Inserm 1052, CNRS 5286, Centre Léon Bérard, Cancer Research Center of Lyon, Lyon, France; ^6^ Department of Medical Oncology, Centre Georges François Leclerc, Dijon, France

**Keywords:** radiomics, NSCLC, immunotherapy, PD-L1 inhibitors, transcriptomics

## Abstract

**Introduction:**

In this study, we aim to build radiomics and multiomics models based on transcriptomics and radiomics to predict the response from patients treated with the PD-L1 inhibitor.

**Materials and methods:**

One hundred and ninety-five patients treated with PD-1/PD-L1 inhibitors were included. For all patients, 342 radiomic features were extracted from pretreatment computed tomography scans. The training set was built with 110 patients treated at the Léon Bérard Cancer Center. An independent validation cohort was built with the 85 patients treated in Dijon. The two sets were dichotomized into two classes, patients with disease control and those considered non-responders, in order to predict the disease control at 3 months. Various models were trained with different feature selection methods, and different classifiers were evaluated to build the models. In a second exploratory step, we used transcriptomics to enrich the database and develop a multiomic signature of response to immunotherapy in a 54-patient subgroup. Finally, we considered the HOT/COLD status. We first trained a radiomic model to predict the HOT/COLD status and then prototyped a hybrid model integrating radiomics and the HOT/COLD status to predict the response to immunotherapy.

**Results:**

Radiomic signature for 3 months’ progression-free survival (PFS) classification: The most predictive model had an area under the receiver operating characteristic curve (AUROC) of 0.94 on the training set and 0.65 on the external validation set. This model was obtained with the *t*-test selection method and with a support vector machine (SVM) classifier. Multiomic signature for PFS classification: The most predictive model had an AUROC of 0.95 on the training set and 0.99 on the validation set. Radiomic model to predict the HOT/COLD status: the most predictive model had an AUROC of 0.93 on the training set and 0.86 on the validation set. HOT/COLD radiomic hybrid model for PFS classification: the most predictive model had an AUROC of 0.93 on the training set and 0.90 on the validation set.

**Conclusion:**

In conclusion, radiomics could be used to predict the response to immunotherapy in non-small-cell lung cancer patients. The use of transcriptomics or the HOT/COLD status, together with radiomics, may improve the working of the prediction models.

## Introduction

Over the last few years, immune checkpoint inhibitors (ICI) targeting the PD-1 pathway have changed the prognosis and survival of patients treated for advanced non-small-cell lung cancer (NSCLC). PD-1/PD-L1 inhibitors are being increasingly used as a standard of care in first- and sometimes second-line therapies, particularly when there is no targetable oncogenic addiction (1–4). However, not all patients will benefit from a response to ICI, and biomarkers are needed to select the patient most likely to benefit from those treatments to improve treatment efficacy, decrease treatment-associated costs, and prevent toxicities (5, 6).

The PD-L1 status is currently used to select patients who will be treated with ICI. In the first-line setting, pembrolizumab is now a standard of care in PD-L1-positive (≥50%) NSCLC (7), while combinations of pembrolizumab or atezolizumab with chemotherapy have shown their superiority over chemotherapy alone, irrespective of PD-L1 expression level (8–10). However, an assessment of PD-L1 expression through immunohistochemical staining is challenging since the threshold for positive PD-L1 labeling on tissue samples is questionable. In addition, PD-L1 expression shows spatial and temporal variability (11). Furthermore, tumors with an overall activated immune microenvironment marked by a high infiltration of immune cells, CD8 T cells (TCD8) in particular, and interferon (IFN)-gamma activation have been described to be more likely to respond to immunotherapy. This has led our group to report a HOT status based on a 27-gene expression–based signature (12, 13).

In parallel, radiomics is a recent discipline that is being increasingly used to determine imaging biomarkers (14). It shows great potential in oncology in patient stratification as well as in predicting the tumor response to treatments (15, 16), overall survival, and the phenotype of tumors (17, 18). Radiomics has been used to predict response to anti-PD-L1 immunotherapy and assess tumor-infiltrating CD8 cells or CD3 cells (18). Consequently, radiomics appears promising in the development of biomarkers of tumor response to PD-1/PD-L1 inhibitors as well as HOT/COLD status prediction. The aim of this study is to develop a radiomic model from pretherapeutic computed tomography (CT) to predict disease control at 3 months in patients treated with nivolumab, pembrolizumab, or atezolizumab in the second- or third-line treatment of stage IV NSCLC. In this study, we also aim to build multiomic models on the basis of transcriptomics and radiomics to predict disease control at 3 months in patients treated with the PD-L1 inhibitor and to predict the HOT/COLD tumor status.

## Materials and methods

### Patient selection and data collection

Eligible patients were those presenting with previously treated histology-proven advanced NSCLC and who had received at least one cycle of either nivolumab, pembrolizumab, or atezolizumab as a single agent between January 2015 and December 2017 in the Léon Bérard Cancer Center (Lyon, France) and the comprehensive Georges-François Leclerc Cancer Center (Dijon, France). Patient data were collected after institutional review board approval. Patients not agreeing to the use of their clinical data for an academic study were excluded according to national and European laws.

Clinical and pathological data were collected using electronic medical records. Clinical variables included sex, age at ICI initiation, and outcome-related data [progression-free survival (PFS) under ICI, overall survival (OS), radiological tumor response at 3 months (12 weeks), and best radiological response according to RECIST 1.1].

To build the models, patients were divided into two classes. The first class was made up of patients who showed complete response (CR), partial response (PR), or stable disease (SD) at 3 months and were considered patients with disease control (DC). The second class was made up of patients with progressive disease (PD) according to RECIST 1.1 and/or clinical progression or death before 3 months.

The patients included in the study underwent a CT scan with available DICOM images 1 month prior to the beginning of the treatment at most.

The data cutoff date was February 2, 2019.

### Patient inclusion

Among the 160 patients treated for NSCLC in Lyon with nivolumab, pembrolizumab, or atezolizumab as a single agent as second- or third-line therapies between January 2015 and December 2017, 110 patients (60 DC and 50 PD) had exploitable DICOM images and 51 had both genomics and imaging data.

Among the 118 patients treated in Dijon, 85 patients (61 DC and 24 PD) had exploitable DICOM images and three had both transcriptomic and imaging data. Patient characteristics are summarized in Table 1.

**Table 1:** Patient characteristics in each dataset.

Patients	Lyon (*n* = 110)	Dijon (*n* = 85)
Gender, *n* (%)		
Female	39 (35.5)	24 (28.2)
Male	71 (64.5)	61 (71.7)
Age: mean (range)	61.7 (36.5–85)	64.3 (37.7–83.5)
Histological subtypes, *n* (%)		
Adenocarcinoma	77 (70)	49 (57)
Squamous cell	21 (19)	36 (43)
Other	13 (11)	0
The stage at diagnosis, *n* (%)		
II	4 (3)	10 (12)
III	16 (15)	19 (22)
IV	90 (82)	56 (66)
Performance status, *n* (%)		
0	12 (11)	30 (35)
1	68 (62)	36 (42)
2	30 (27)	19 (22)
PD-L1 expression, *n* (%)		
0%	23 (21)	23 (27)
1%–49%	34 (31)	17 (20)
≥50%	17 (15)	9 (11)
Not available	36 (33)	36 (42)
Molecular alterations, *n* (%)		
EGFR	7 (6)	4 (5)
KRAS	37 (34)	20 (24)
Other	7 (6)	4 (5)
None	51 (46)	57 (52)
Progression-free survival (months): mean (range)	7.4 (0.2–39.1)	4.9 (0.2–51.7)
Radiological tumor response at 3 months (12 weeks)	PD = 61	PD = 59
	PR = 13	PR = 7
	SD = 34	SD = 16
		CR = 1

### Transcriptomics

In a 54-patient subgroup with formalin-fixed paraffin-embedded samples, we retrieved targeted-RNA sequencing data previously reported by our group (GSE161537) (19, 20).

Each tumor was classified as HOT or COLD based on a 27-gene expression signature. HOT tumors were shown to be characterized by an overall activated immune microenvironment by (i)-PD-L1 and IDO1 expression, (ii)-TCD8 infiltrate, and (iii) activation of the IFN-gamma pathway. Among the 54 patients, 31 and 23 patients had tumors classified as HOT or COLD, respectively.

### Radiomic feature extraction

Patients underwent CT scans using various systems [Siemens (*n* = 63), Philips (*n* = 25), General Electric (*n* = 85), Toshiba (*n* = 9), Hitachi (*n* = 2)] with various protocols (voltage range: 100–130 kV, X-ray-tube current: 350–700 mAs, pitch: 0.8–1.5). Images were reconstructed using a soft kernel for all patients [range of image thickness (1–3 mm)].

**Figure 1:**
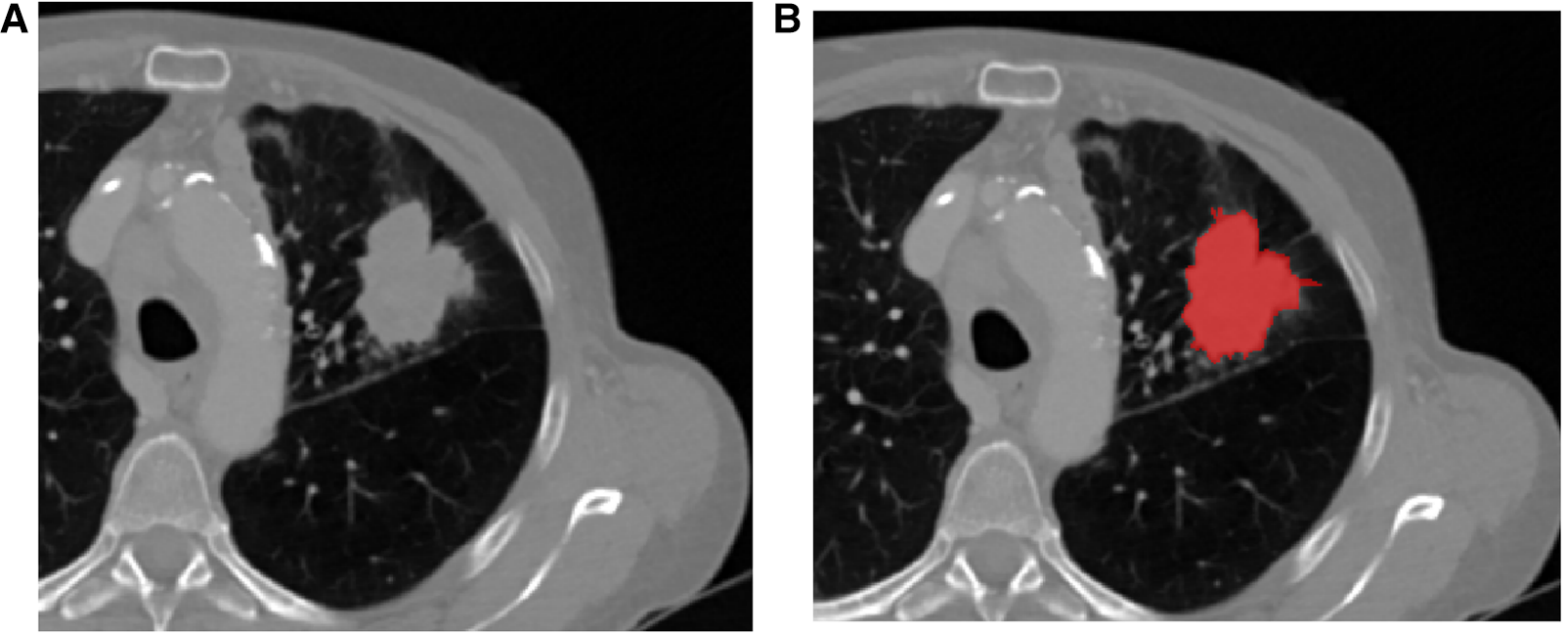
(**A**) Lung adenocarcinoma of the upper left lobe with spiculated margins. During the segmentation step (**B**), the radiologist defined the contours of the tumor, selecting all the tissue parts and excluding peripheral vessels or non-tumoral lung condensation. The segmentation is performed in three dimensions.

Images were automatically loaded on an in-house software developed on MATLAB R2019a (The Mathworks, Natick, MA, USA). The tumor was manually segmented in three dimensions by an experienced radiologist (AB, nine years of experience in oncology imaging), and the data were blinded for clinical results. Tumor segmentation was performed slice by slice to generate the tumor mask using ITK-SNAP (www.itksnap.org). The radiologist defined the contours of the tumor on the soft-kernel reconstruction images (Figure 1). If large vessels or adjacent organs were infiltrated by the tumor, they were included in the mask. The primary tumor was preferentially segmented, but if the patient had undergone prior surgery for the tumor and was treated for recurrence, the largest lung or mediastinal tumor was included in the study.

**Figure 2:**
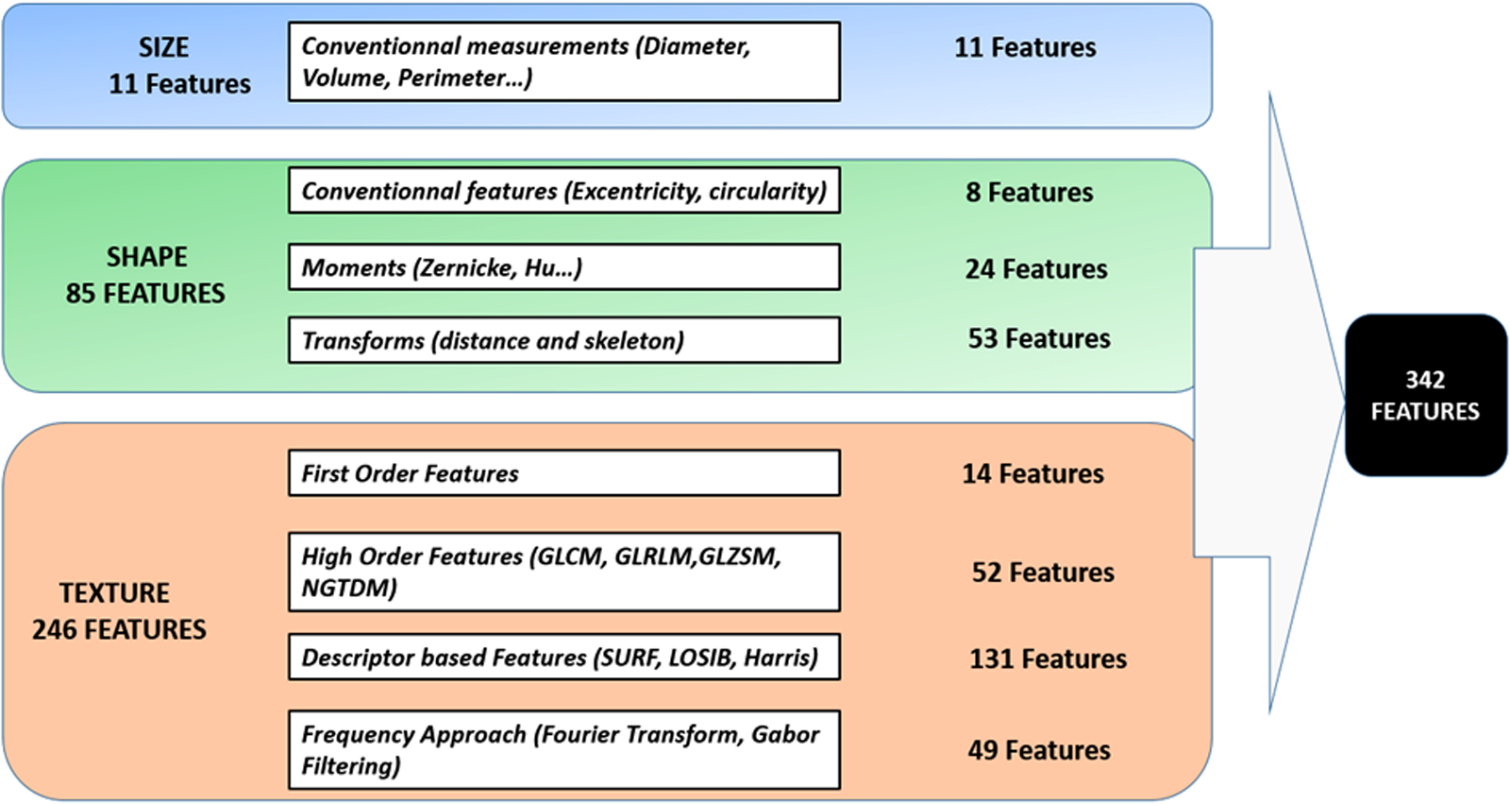
List of radiomic features. Radiomic features include size features, shape features, and texture features [image-based first-order (or histogram) features, high-order features based on different texture matrices or descriptors, and frequency-domain characteristics].

Three hundred and forty-two radiomic features were extracted according to Bouhamama et al. (21). The full list of features is summarized in Figure 2.

Size and shape features were directly extracted from the binary mask. Intensity distribution features were extracted from the masked MR images and from the histogram built using 256 bins.

Before the extraction of texture features, voxels were resampled to be isotropic using an affine transformation and a nearest-neighbor interpolation and discretized to a smaller number of gray levels. This operation was done using an equal-probability algorithm to define decision thresholds in the volume; for instance, the number of voxels for a given reconstructed level was the same in the quantized volume for all gray levels. Images were discretized to 8, 16, 24, 32, 40, 48, and 64 grey levels to build four texture matrices; GLCM and GLRLM were computed for four directions (0°, 45°, 90°, and 135°) with an offset of one pixel. For GLSZM and NGTDM, 26-pixel connectivity was used. For the Gabor characteristics, filter responses were computed at different scales (*n* = 5), different orientations (*n* = 6), and with a minimum wavelength of three.

### Feature selection

**Figure 3:**
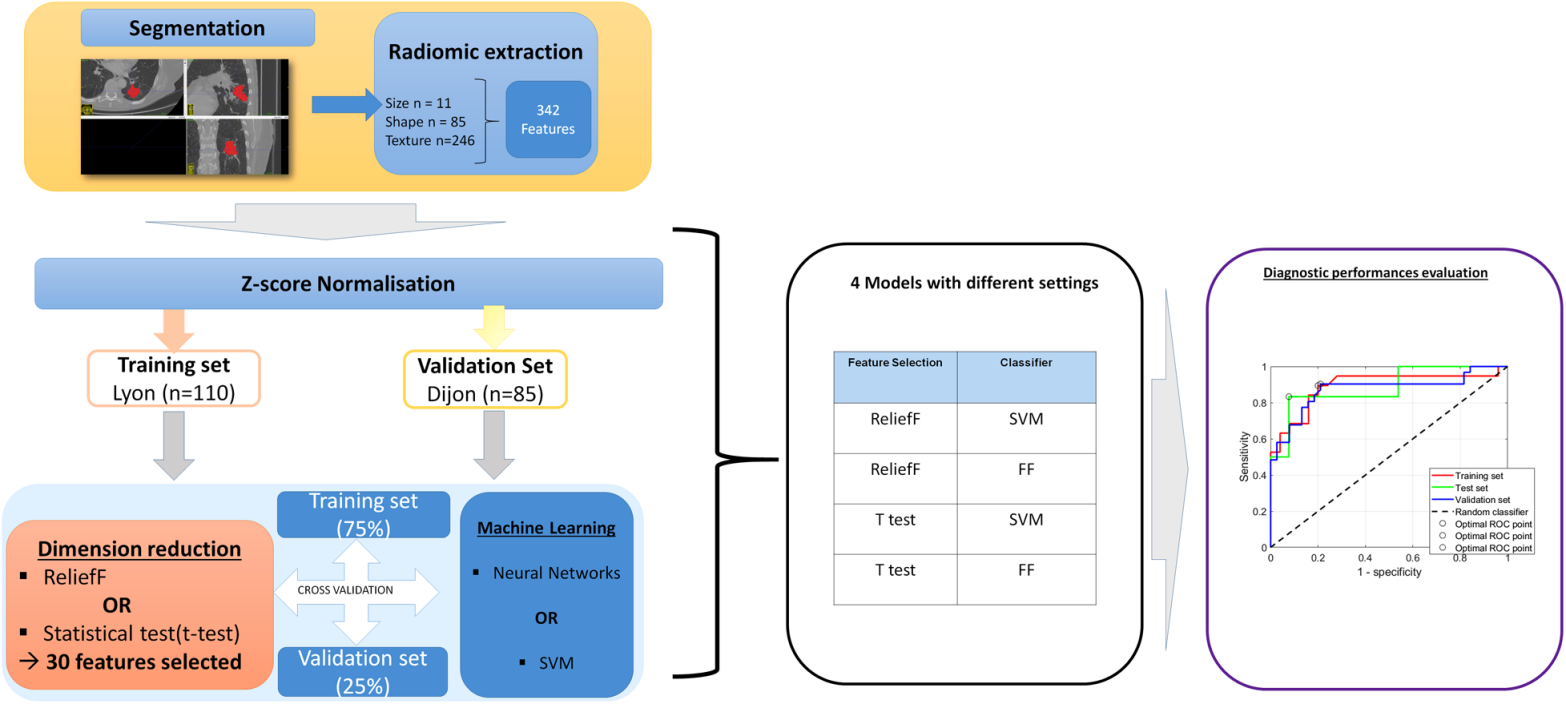
Radiomic analysis pipeline for the prediction model of the 3-month PFS, using the Lyon cohort for training and the Dijon cohort for validation. During the segmentation step, the radiologist defined the contours of the tumor, selecting all the tissue parts and excluding peripheral vessels or non-tumoral lung condensation. In total, 342 radiomic features were automatically extracted. Each database was separately normalized using the *z*-score. The training set was used to build the model. Dimension reduction was performed using one of two feature-selection methods (*t*-test or ReliefF). Then, machine learning was performed using two different classifiers: an SVM or an artificial neural network with feed-forward (FF) multilayer perceptron architecture. Internal validation was systematically performed to evaluate overfitting, using a hold-out cross-validation technique, with 75% of the database used for training and 25% for validation. Then, the model inference was performed, using the different validation sets, and the performance was evaluated by receiver operating characteristic (ROC) analysis. Finally, eight models were created with different settings.

**Figure 4:**
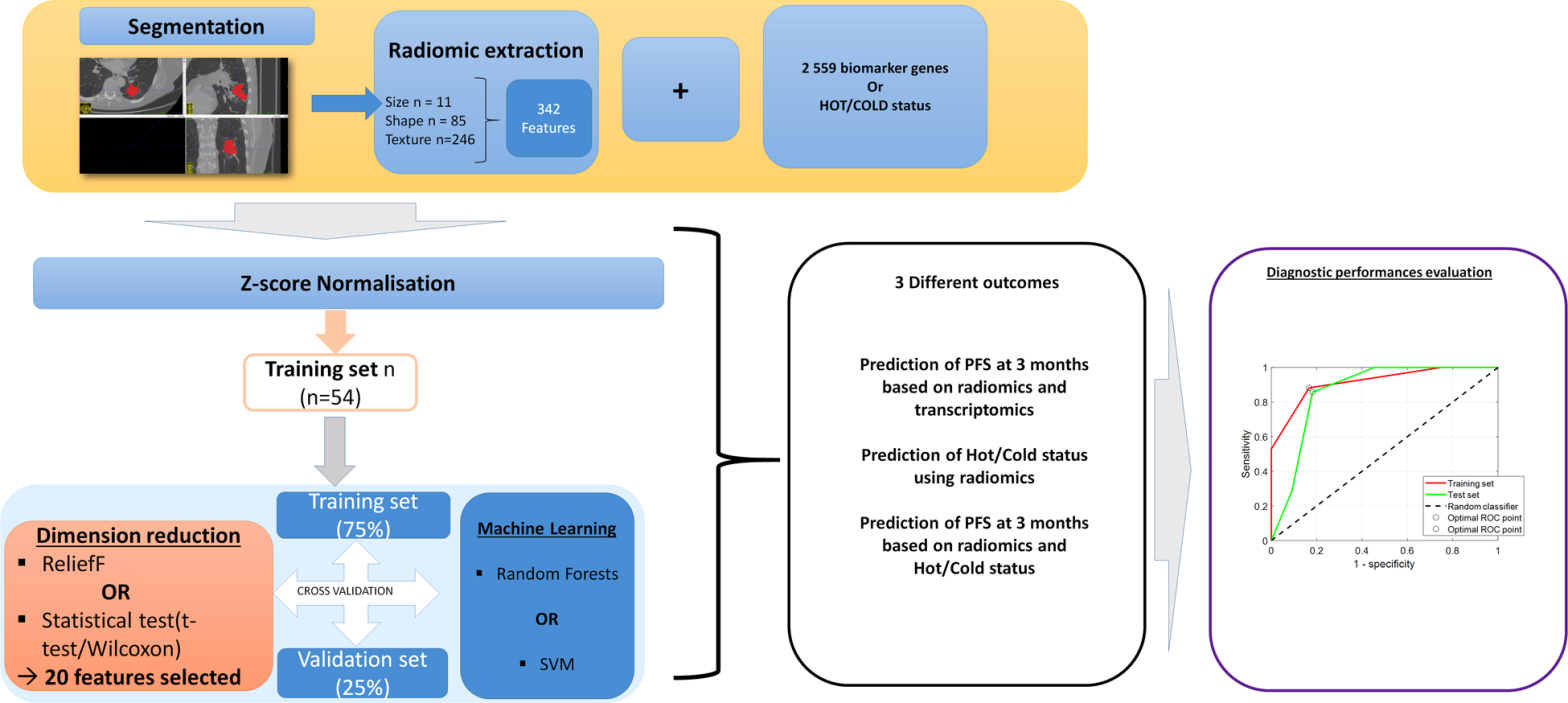
Analysis pipeline for the multiomic models. The segmentation step and extraction of features are the same as those presented in Figure 1. Since the number of patients is reduced to 54, it was not possible to build a validation set and only the cross-validation step was performed. Depending on the outcome to be predicted, the radiomic features were combined with genomics or the HOT/COLD status. Dimension reduction was performed using one of two feature-selection methods (*t*-test or Wilcoxon test or ReliefF). Then, machine learning was performed with two different classifiers: an SVM or random forest, with a split of 10. Internal validation was systematically performed to evaluate overfitting, using a hold-out cross-validation technique, with 75% of the database used for training and 25% for validation. The performance was evaluated by ROC analysis. Finally, eight models were created with different settings.

After the extraction of radiomic features, each database was separately normalized using the Z-score. An initial step of dimensionality reduction was performed (Figures 3, 4). Two different approaches were tested. In the first approach, feature selection was performed using the ReliefF algorithm, with *k* = 10 being the nearest neighbor. In the second approach, we used a statistical method accounting for relevancy and redundancy. The method ranks the features by computing a score combining the results of a statistical test *Z* (for relevancy) and correlation information to outweigh the *Z*-value of potential features (for redundancy) using

S=Z×[(1−α)ρ]

where *ρ* is the average absolute value of the cross-correlation coefficient between the candidate feature and all previously selected features; *α* is the weighting factor—fixed here at 0.7. Different statistical tests were evaluated to compute the *Z*-value: the *t*-test, Wilcoxon test, and AUROC.

The number of features integrated into the model was adjusted to the size of the data so that it was consistent with the number of observations. This number of features is further detailed for each model.

### Predictive model training

Various models were trained with different databases, different outcomes, and different combinations of feature selection methods and classifiers. In each case, we performed a binary classification (DC vs. PD or HOT vs. COLD). We compared two different classifiers for each model (convolutional neural network (CNN) vs. SVM).

For the model trained on radiomic data, since the number of patients was more than 100, we used an artificial neural network with a feed-forward multilayer perceptron architecture. For the three other models (trained on radiogenomic data), the number of patients was smaller, and we used random forests with a split of 10. For every model, we also used a support vector machine trained with a linear kernel and box constraints set to one as a second classifier. The following predictive models were built.

#### Prediction of PFS at 3 months based on radiomics

To build this model, two classes were considered. The first class constituted patients who showed CR, PR, or SD at 3 months and were considered patients with DC. The second class constituted patients with PD according to RECIST 1.1 and/or clinical progression or death before 3 months. The training database was built using the patients treated in Lyon [*n* = 110 patients (60 DC vs. 50 PD)]. The number of selected features after feature reduction was set at *n* = 30. To evaluate overfitting, a hold-out cross-validation technique was performed with 75% of the database used for training and 25% for validation.

Next, the model inference was performed separately on the Dijon database used as an external validation set [*n* = 85 patients (61 DC and 24 PD)]. The diagnostic performance metrics [area under the receiver operating characteristic curve (AUROC), accuracy, sensitivity, specificity, misclassification rate, and misclassified patients] were measured for each dataset and then iteratively compared to adjust the number of features embedded in the model (Figure 3).

#### Prediction of PFS at 3 months based on radiomics and genomics

Since the number of patients who had both radiomic data and genomic data was lower than in the previous step, 51 patients treated in Lyon and three patients treated in Dijon were merged into a single cohort (39 DC and 15 PD). The population was dichotomized into two classes of DC and PD as previously. For each patient, the 342 radiomic features and 2,559 oncology-related biomarker genes were merged into a single database. The number of selected features after dimension reduction was set at *n* = 20. To evaluate overfitting, a hold-out cross-validation technique was performed with 75% of the database used for training and 25% for validation (Figure 4).

#### Prediction of the HOT/COLD status using radiomics

To build this model, the 54 patients who had both radiomic and genomic data available were included. The population was dichotomized into two classes of HOT status and COLD status, as previously explained. For each patient, the 342 radiomic features were included in the database. The number of selected features after dimension reduction was set at *n* = 15. To evaluate overfitting, a hold-out cross-validation technique was performed with 75% of the database used for training and 25% for validation (Figure 4).

#### Prediction of PFS at 3 months based on radiomics and HOT/COLD status

To build this model, the 54 patients who had both radiomic data and HOT/COLD status were included. The population was dichotomized into two classes of DC (*n* = 33) and PD (*n* = 21). For each patient, the 342 radiomic features and the HOT/COLD status were merged into a single 343-feature database. The number of selected features after dimension reduction was set at *n* = 15 (including the HOT/COLD statuses). To evaluate overfitting, a hold-out cross-validation technique was performed with 75% of the database used for training and 25% for validation (Figure 4).

## Results

### Patient survival

**Figure 5:**
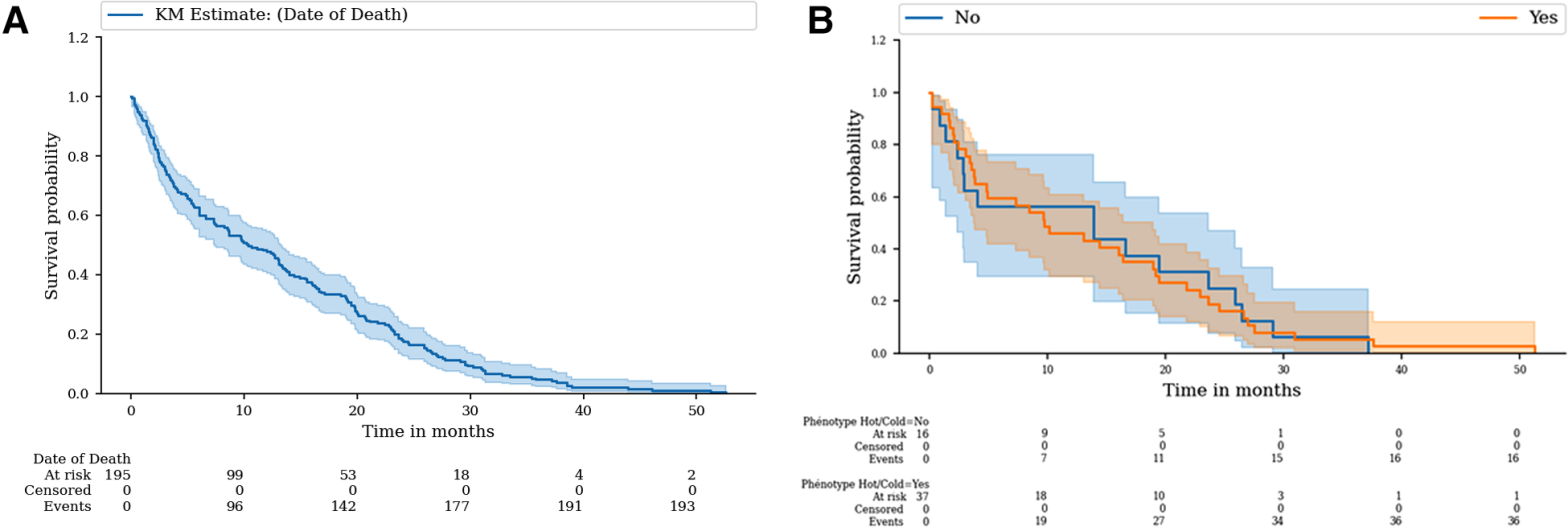
Kaplan–Meier curves of (**A**) overall survival in the whole cohort, (**B**) progression survival of the patients treated in Dijon and Lyon, and (**C**) progression survival of HOT tumors (“Yes”) patients vs. COLD tumor patients (“No”).

The PFS of the whole cohort was 36.9% (95% CI: 30.2%–43.7%) at 3 months and 24.1% (95% CI: 18.4%–30.3%) at 6 months. The mean PFS was 63 days. The OS of the whole cohort was 75.4% (95% CI: 68.7%–80.8%) at 3 months and 61.5% (95% CI: 54.3%–68.0%) at 6 months. The median OS was 314 days. There was no difference between the PFS of the Lyon patients and the Dijon patients (*p* = 0.995). With regard to the HOT/COLD status, there was no difference between the PFS of HOT tumor patients and that of the cold tumor patients (*p* = 0.199). Kaplan–Meier survival curves are shown in Figure 5.

### Diagnostic performance of the predictive models

#### Prediction of PFS at 3 months based on radiomics

**Figure 6:**
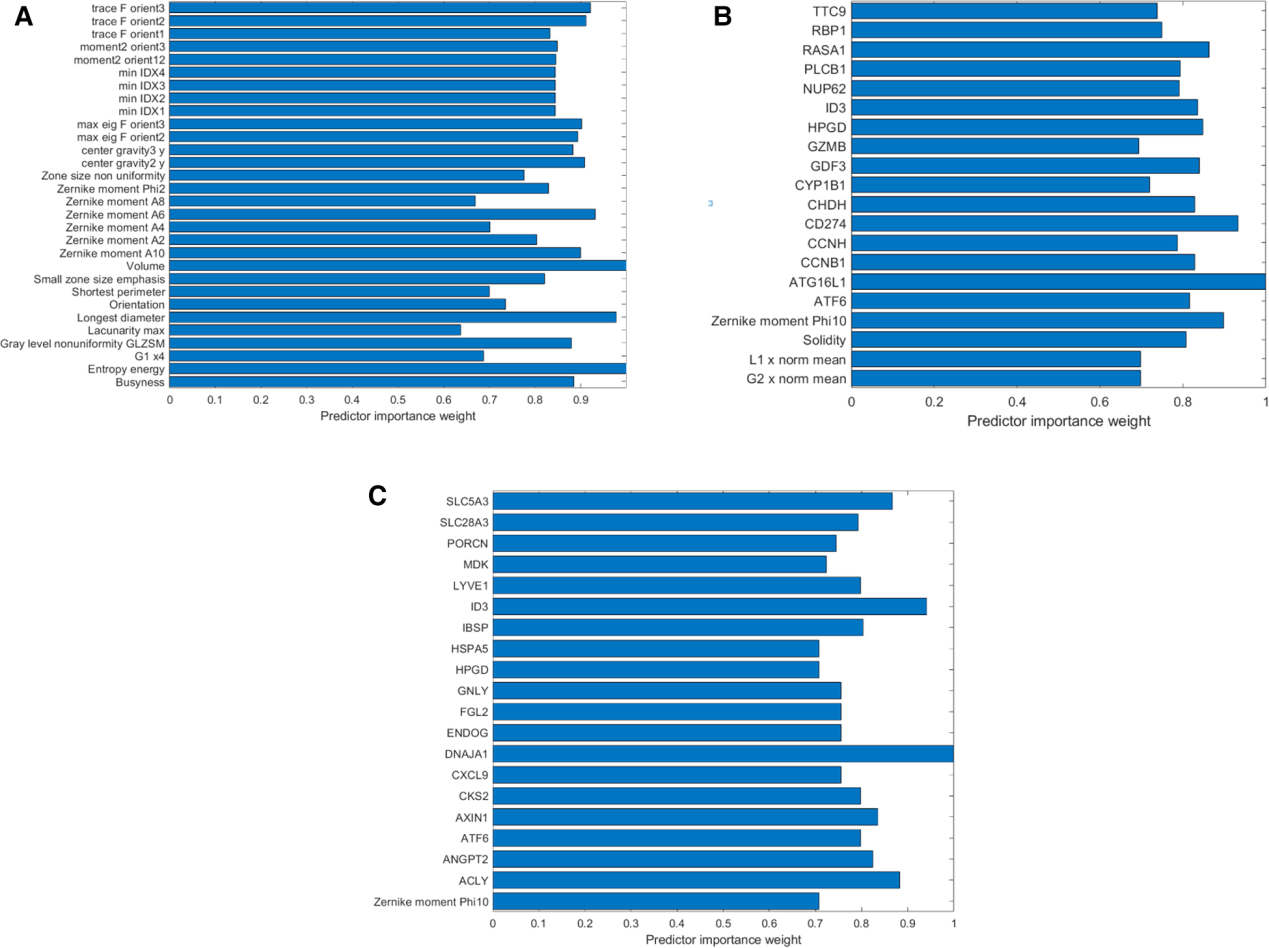
List of features according to their predictor importance weight. Thirty features were selected using the *t*-test and included in the first predictive model (progression-free survival at 3 months). (**B,C**) Features selected to train the second predictive model of PFS at 3 months, based on radiomics and genomics, after feature selection using the ReliefF algorithm and Wilcoxon test, respectively. Sixteen genes and four radiomic features were selected using the ReliefF algorithm (**B**); and 19 genes and one radiomic feature using the statistical test (**C**). Note that when using ReliefF, the gene encoding for PD-L1, CD274, was selected among the 2,559 genes tested, and when using the Wilcoxon test, gene CXCL9 was included. Those two genes are described as common and prominent biomarkers of response to immunotherapy. Comparing the two selection methods, one Zernike moment was selected by the two techniques, and two genes (ID3 and ATF6) were selected by both methods, highlighting that some shape features and some genes may be used as biomarkers of the clinical response to immunotherapy.

Two different methods of feature selection were attempted and combined with two different classifiers, resulting in four different models. A list of these features is summarized in Table 2. Features with their respective weight of predictor importance are listed in Figure 6A.

**Table 2:** List of the features included in each model after the dimension reduction step.

Prediction model	Feature selection method			
1- Prediction of PFS at 3 months based on radiomics	*t*-test		ReliefF	
	21 shape features: Size (*n* = 1), Hu moments (*n* = 8), affine moments (*n* = 6), Skelet features (*n* = 4), Zernike features (*n* = 2). 9 Texture Features: first order (*n* = 3), GLRLM (*n* = 2), GLZLM (*n* = 1), NGTDM (*n* = 1), SURF features (*n* = 1), Harris features (*n* = 1)		16 shape features: size (*n* = 4), Zernike features (*n* = 6), dist features (*n* = 4), skelet features (*n* = 2) 14 Texture Features: first order (*n* = 1), GLZLM (*n* = 3), NGTDM (*n* = 1), Fourier transform (*n* = 7), lacunarity (*n* = 1)	
2- Prediction of PFS at 3 months based on radiomics and transcriptomics	*t*-test	Wilcoxon	AUROC	ReliefF
	– 1 shape feature: Zernike features (*n* = 1) – 19 genes	– 1 shape feature: Zernike features (*n* = 1) – 19 genes	– 1 shape feature: Zernike features (*n* = 1) – 19 genes	– 2 shape feature: size (*n* = 1), Zernike features (*n* = 1) – 2 Texture Features: grad features (*n* = 2) – 16 genes
3- Prediction of HOT/COLD status using radiomics	*t*-test	Wilcoxon	AUROC	ReliefF
	– 3 shape features: size (*n* = 3) – 12 Texture Features: first order (*n* = 1), GLCM (*n* = 1), GLZLM (*n* = 3), Fourier transform (*n* = 2), grad features (*n* = 2), Losib features (*n* = 2)	– 4 shape features: size (*n* = 4) – 11 Texture Features: first order (*n* = 2), GLZLM (*n* = 5), grad features (*n* = 3), Losib features (*n* = 1)	– 6 shape features: size (*n* = 3), Affine moment features (*n* = 3) – 9 Texture Features: first order (*n* = 1), GLZLM (*n* = 4), grad features (*n* = 3), Losib features (*n* = 1)	– 9 shape features: size (*n* = 2), Zernike features (*n* = 1), dist features (*n* = 6) – 6 Texture Features: GLRLM (*n* = 3), GLZLM (*n* = 1), Fourier transform (*n* = 2)
4- Prediction of PFS at 3 months based on radiomics and HOT/COLD status	*t*-test	Wilcoxon	AUROC	ReliefF
	– 14 shape features: Size (*n* = 1), Zernike features (*n* = 3), dist features (*n* = 9), skelet features (*n* = 1) – HOT/COLD status	– 9 shape features: size (*n* = 1), Zernike features (*n* = 2), skelet features (*n* = 6) – 5 Texture Features: grad features (*n* = 3), Losib features (*n* = 2) – HOT/COLD status	– 8 shape features: Zernike Features (*n* = 2), skelet features (*n* = 2), affine moment features (*n* = 1), dist features (*n* = 3) – 6 Texture features: Grad features (*n* = 3), Losib features (*n* = 2), Fourier transform (*n* = 1) – HOT/COLD status	– 4 shape features: size (*n* = 1), Zernike features (*n* = 3) – 10 texture features: GLRLM (*n* = 6), Fourier transform (*n* = 4) – HOT/COLD status

Thirty features were selected using the ReliefF algorithm:

– 16 shape features: size (*n* = 4), Zernike features (*n* = 6), dist features (*n* = 4), and skelet features (*n* = 2)
– 14 texture features: first order (*n* = 1), GLZLM (*n* = 3), NGTDM (*n* = 1), Fourier transform (*n* = 7), and lacunarity (*n* = 1)

Thirty features were selected using the *t*-test selection method:

– 21 shape features: size (*n* = 1), Hu moments (*n* = 8), affine moments (*n* = 6), skelet features (*n* = 4), and Zernike features (*n* = 2)
– 9 texture features: first order (*n* = 3), GLRLM (*n* = 2), GLZLM (*n* = 1), NGTDM (*n* = 1), SURF features (*n* = 1), and Harris features (*n* = 1)

**Figure 7:**
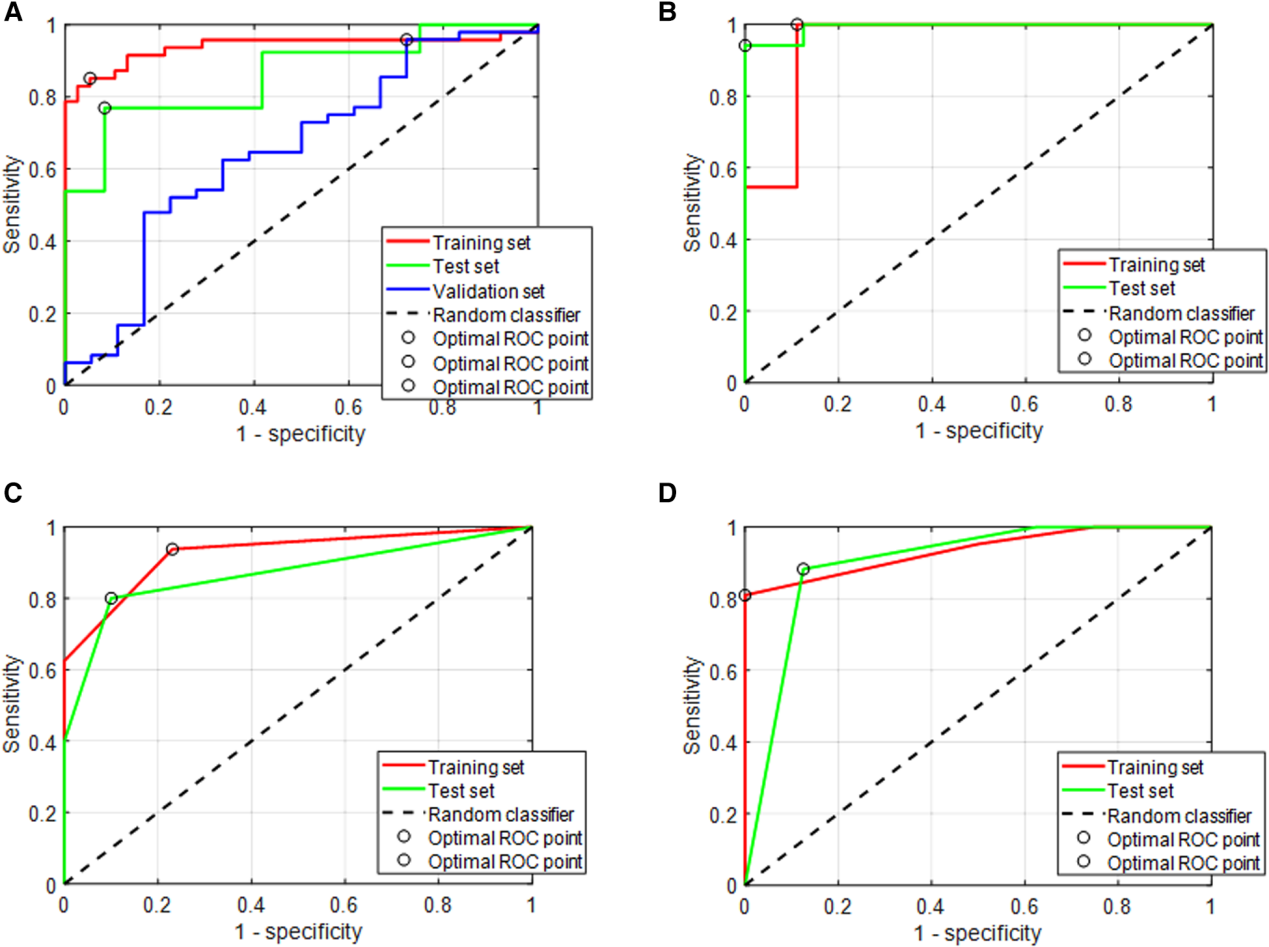
ROC analysis of four different models. (**A**) The Lyon population was used as a training set. A *t*-test was used to select the 30 most relevant features and an SVM was used as the classifier to classify patients with DC and PD. Then, the model inference was performed using the Dijon validation set. The area under the ROC curve (AUC) in red shows the performance on the training set (green for the cross-validation set); the curves in blue show the performance on the Dijon validation cohort. (**B**) The model was built with the 54 patients who had both radiomic and genomic data. A *t*-test was used to select the 20 most relevant features among 342 radiomic features and 2,559 genes, and an SVM was used as the classifier to classify patients with a DC and PD. The AUC in red shows the performance on the training set; the AUC in green is intended for the cross-validation set. It was not possible to build a validation set since the number of patients was lower than in procedure A. (**C**) The same 54 patients as in B were included in this study. The 20 most relevant radiomic features were selected using the *t*-test as a selection method and decision trees using a split of 10 were used as the classifier. The aim of this study was to use radiomics to predict the HOT/COLD status. (**D**) This model was built by merging radiomic features with the HOT/COLD status. The 19 most relevant features were selected, using their AUROC among 342 radiomic features, and the HOT/COLD status was added. Decision trees using a split of 10 were used to classify patients with DC and PD.

The most predictive model had an AUROC of 0.94, a sensitivity of 88.2%, and a specificity of 85.1% on the training set, which were, respectively, 0.65, 95.8%, and 27.8% on the external validation set. This model was obtained with the *t*-test selection method and with an SVM classifier. The performances of the four different models are summarized in Table 3, and the AUC is presented in Figure 7A.

**Table 3:** Diagnostic performance for the training set on the Lyon dataset and external validation set on the Dijon dataset for each classifier; feature selection method for the prediction of PFS at 3 months based on radiomics.

		Training set (Lyon)								Validation set (Dijon)					
Reduction method	Learning method	AUC	Accuracy (%)	Sensitivity (%)	Specificity (%)	AUC (test)	Accuracy (%) (test)	Sensitivity (%) (test)	Specificity (%) (test)	AUC	Accuracy (%)	Sensitivity (%)	Specificity (%)	Mis-classification rate (%)	No. misclassified patients
ReliefF	Neural network	0.88	85.9	82.3	92.2	0.88	76	76.9	91.7	0.46	60	96.7	25	40.0	34
T-test	Neural network	0.81	85.9	100.0	69.3	0.78	68	92.9	54.5	0.52	50.6	97.9	27.8	49.4	30
ReliefF	SVM	0.94	92.9	89.3	97.4	0.90	76	71.4	100.0	0.63	48.2	97.9	22.2	51.8	32
T-test	SVM	0.94	88.2	85.1	94.7	0.86	80	76.9	91.7	0.65	49.4	95.8	27.8	50.6	31

#### Prediction of PFS at 3 months based on radiomics and genomics

During the feature selection, 16 genes and four radiomic features were selected using the ReliefF algorithm and 19 genes and one radiomic feature were selected using statistical tests. A list of these features is summarized in Table 2. Features with their respective weights of predictor importance are listed in Figures 6B and C.

The most predictive model had an AUROC of 0.95, a sensitivity of 87.1%, and a specificity of 100% on the training set; which were respectively 0.99, 94.1%, and 100% on the validation set. This model was obtained by combining the *t*-test selection method and an SVM as a classifier. The performances of the eight different models are summarized in Table 4, and the AUC of the best model is presented in Figure 7B.

**Table 4:** Diagnostic performances for (i) the prediction of PFS at 3 months based on radiomics and genomics, (ii) prediction of the HOT/COLD status using radiomics, and (iii) prediction of PFS at 3 months based on radiomics and HOT/COLD status. Since only 54 patients had both genomic and radiomic data, the validation of the models was made with hold-out cross-validation.

			Training set				Validation set (cross-validation)					
	Reduction method	Learning method	AUC	Accuracy (%)	Sensitivity (%)	Specificity (%)	AUC	Accuracy (%)	Sensitivity (%)	Specificity (%)	Mis-classification rate (%)	No. misclassified patients
PFS at 3 months, based on radiomics and transcriptomics	AUROC	Decision trees	1.00	96.8	95.2	100	0.91	80	94.4	57.2	20	6
	RELIEFF	Decision trees	0.94	96.8	100	88.9	0.91	88	82.3	100	12	3
	*t*-test	Decision trees	0.98	96.8	95.2	100	0.85	84	83.3	85.7	16	4
	Wilcoxon	Decision trees	0.99	96.8	95.5	100	0.92	80	94.1	62.5	20	6
	AUROC	SVM	0.97	90.3	100	80.0	0.95	84	94.4	85.7	16	4
	RELIEFF	SVM	0.95	80.6	95.2	100	0.93	84	83.3	100	16	4
	*t*-test	SVM	0.95	87.1	100	88.9	0.99	84	94.1	100	16	4
	Wilcoxon	SVM	0.98	93.5	95.5	88.9	0.95	92	100	75	8	2
HOT/COLD status using radiomics	AUROC	Decision trees	0.93	86.2	93.8	79.3	0.87	84	80	90	16	4
	RELIEFF	Decision trees	0.81	79.3	81.2	76.9	0.82	72	80	90	28	8
	*t*-test	Decision trees	0.93	86.2	80.0	90.0	0.87	84	80	90	16	4
	Wilcoxon	Decision trees	0.92	86.2	88.3	83.3	0.86	84	85.7	81.8	16	4
	AUROC	SVM	0.81	79.3	94.1	66.7	0.86	60	85.7	91.1	40	12
	RELIEFF	SVM	0.81	75.9	100	53.8	0.81	76	86.7	60	24	7
	*t*-test	SVM	0.80	69.0	68.8	92.4	0.84	68	86.7	80	32	9
	Wilcoxon	SVM	0.81	72.4	100	53.8	0.85	68	86.7	90	32	9
PFS at 3 months, based on radiomics and HOT/COLD status	AUROC	Decision trees	0.93	86.2	81.0	100	0.90	88	88.2	87.5	12	3
	RELIEFF	Decision trees	0.93	93.1	100	0.0	0.50	60	60	40	40	12
	*t*-test	Decision trees	0.90	86.2	85.7	87.5	0.85	72	100	50	28	8
	Wilcoxon	Decision trees	0.91	82.8	80.0	88.9	0.88	84	83.3	85.7	16	4
	AUROC	SVM	0.88	79.3	90.5	75.0	0.87	84	82.4	87.5	16	4
	RELIEFF	SVM	0.83	79.3	100	66.7	0.82	76	94.4	42.9	24	7
	*t*-test	SVM	0.86	79.3	80.0	88.9	0.86	76	88.9	71.5	24	7
	Wilcoxon	SVM	0.88	82.8	95.2	75.0	0.87	76	100	50	24	7

#### Prediction of HOT/COLD status using radiomics

A list of the features included in the models is summarized in Table 2.

The most predictive model had an AUROC of 0.93, a sensitivity of 86.2%, and a specificity of 88.3% on the training set, which were, respectively, 0.86, 84%, and 80% on the validation set. This model was obtained by using a *t*-test as a selection method and with decision trees as a classifier. The performances of the eight different models are summarized in Table 4, and the AUC of the best model is presented in Figure 7C.

#### Prediction of PFS at 3 months based on radiomics and HOT/COLD status

A list of the features included in the models is summarized in Table 2.

The most predictive model had an AUROC of 0.93, a sensitivity of 86.2%, and a specificity of 81% on the training set, which were, respectively, 0.90, 88%, and 88.2% on the validation set. This model was obtained with AUROC as a selection method and with decision trees as a classifier. The performances of the eight different models are summarized in Table 4, and the AUC of the best model is presented in Figure 7D.

## Discussion

In this work, we have demonstrated that radiomics extracted from pretherapeutic CT scans were useful for predicting different clinical outcomes such as response to treatment and the HOT/COLD status in NSCLC.

Size and shape features were highly represented in the list of selected features while performing the dimensionality reduction step. Patients with a higher tumor volume had a worse prognosis. This finding shows how prominent the tumor volume is for the prognosis, but it may be an important source of bias, and it is no surprise that patients with advanced cancer had a shorter PFS. However, it may show how relevant feature selection methods are. Therefore, a discussion on a better selection of the patients included in a further study is warranted and different prediction models may be designed for different ranges of tumor sizes. Here, the number of subjects has restricted the creation of various subgroups.

In our study, among the radiomic features included in the model, contrast NGTDM and Gray Level Non-Uniformity had lower values for patients responding to immunotherapy, on the one hand, and a higher value of Long Run High Gray Level Emphasis, on the other hand. This means that tumors that will respond to immunotherapy were more homogeneous than tumors that did not respond to immunotherapy, and those that had a coarse texture had higher runs of high gray level, meaning a higher contrast enhancement. Some other features showed different behaviors, such as Large Zone Size Emphasis, which had higher values in PR patients, or Zone Size Non-Uniformity, which was lower in PR patients, but most features showed more homogeneity in GR patients.

The texture features selected in the prediction model of the HOT/COLD status have shown interesting findings. For example, Gray Level Non-Uniformity, which was selected by both the *t*-test-based and ReliefF methods showed that HOT lesions were more homogeneous. HOT tumors are characterized by a high infiltration of CD8 T cells and GLNU may be correlated with T-cell infiltration, tumor homogeneity, and 3-month PFS.

These results are consistent with those of Sun et al. (18), who reported that lesions with a high CD8 cell score—the more likely to respond to immunotherapy—were the most homogeneous, considering gray-level run-length matrix features. In this study, the authors suggested that homogeneous and hypodense patterns could be representative of inflammatory infiltrates, whereas heterogeneity and high gray levels might be more representative of heterogeneous and intertwined processes, such as chaotic vascularization and necrosis. In contrast, Trebeschi et al. (17) found that lesions with more heterogeneous morphological profiles and non-uniform density patterns were more likely to respond to immunotherapy, irrespective of organ and/or cancer type. However, such different results between Sun et al.’s study, on the one hand, and Trebeshi et al. and our study, on the other hand, are highly disturbing. This enhances the need to create a link between radiomic patterns and tumor phenotype. Indeed, the explanation for the biological phenomenon that leads to heterogeneous imaging or some radiomic patterns is highly hypothetical (22). A better knowledge of the correlation between histology and imaging may help to avoid a misunderstanding of the mechanisms that lead to treatment resistance.

The use of the HOT score in our study may provide some additional data. Indeed, it is known that the HOT score correlates with PD-L1 (23, 24) and IDO1 expression (24) as well as a higher TCD8 infiltration and activation of the IFN-gamma pathway (25). The fact that radiomics can predict the HOT/COLD status is an interesting issue because it implies that the tumor images may reflect specific information about PD-L1 and IDO1 expression as well as TCD8 infiltration and activation of the IFN-gamma pathway. Furthermore, the HOT status is correlated with a better response rate to immunotherapy and better survival. This means that radiomics indirectly predict the 3-month PFS by capturing some phenotypical characteristics in the tumors. This sustains the hypothesis that radiomics may be the link between the microscopic and the macroscopic scales of the tumor (26, 27).

In this study, we have built a multiomic model based on genomics and radiomics. It seems that combining radiomics with genomic data increases the models’ diagnostic performances. Unfortunately, we cannot fairly conclude that the multiomic model outperforms the radiomic model because the number of subjects is significantly lower in the genomic + radiomic group. Indeed, the sequencing of 2,559 oncology-related biomarker genes is not done in current practice, and we lacked genomic data for most patients. The combination of the HOT/COLD status with radiomics also resulted in this model's high performance. This approach is particularly interesting because the HOT/COLD status results from the expression of genes that are predictive of the response to immunotherapy (19). Unfortunately, there was no significant difference in PFS between HOT tumors and COLD tumors in this study, which included a subgroup of patients, but the HOT/COLD status was demonstrated to be predictive of a better response to treatment than in our previous study (19), which included all patients. The transformation of the complete genomic database of 2,559 genes into one single phenotype can be compared with selecting the most relevant features, and this contributes to limiting the risk of overfitting.

However, with regard to some of the genes selected in our models, we may assume that the feature selection method is relevant and is able to capture genomic signatures together with radiomic features. Indeed, using the ReliefF methods—among the total of 20 features, chosen to be included in the models—four were radiomic features and 16 were genomic features. That the genomic features were overrepresented was to be expected because the algorithm has to select features among 2,559 genes and 342 radiomic features. Among genes, CD274 has to be highlighted since this gene encodes PD-L1. As previously explained, PD-L1 currently remains the main biomarker of the immunotherapy response. When the Wilcoxon test was used for the dimensionality reduction step, we did not manage to build a hybrid model since the algorithm selected only one single radiomic feature and 19 genes. However, among those genes, the expression of CXCL9 has to be highlighted. Indeed, CXCL9 is a potential biomarker of immune infiltration (28–31) associated with favorable prognosis in many cancers and has been reported to be one of the most predictive. The fact that this gene was selected from among the 2,559 genes also warrants the consistency of this dimensionality reduction method in our data mining pipeline.

Indeed, tumor size is a bad prognostic factor in itself, and showing that size features are related to a lower PFS indicates no new finding.

The strength of radiomics and imaging is the capacity to study the whole body and the whole tumor volume, whereas biopsies enable the study of only a small sample of tumors. A tumor's spatial heterogeneity is a well-known problem, particularly considering the expression of PD-L1/PD-1 (11). Possibly, radiomics is able to capture the spatial heterogeneity of the tumor. For this reason, it may be challenging to identify correlations between radiomic patterns and histological patterns, unless the radiomic features are correlated to the whole tumor after tumor resection. However, for the same reason, it may be relevant to use radiomics to predict clinical outcomes, particularly for the stratification of patients treated with PD-1/PD-L1 inhibitors. Indeed, only 20%–30% of patients treated with PD-1/PD-L1 inhibitors will show a response to treatment. Although durable responses can occur in patients treated with ICIs (32–34), there is currently no predictive factor of durable response. So, a longer follow-up in a larger cohort may help us create other radiomic predictive models. The emergence of single-photon CT scans is another trail to make more reproducible and more relevant radiomics. Indeed, the ability of single-photon CT scans to provide quantitative data and quantitative maps may help to build the link between physical effects such as photon absorption and the biology of the tumors.

This study has several limitations. First of all, the number of subjects is relatively small. We managed to build a training set with the population from Lyon and an external validation set with the population from Dijon. Although the number of patients treated with PD-L1/PD-1 inhibitors is increasing, the use of ICI is relatively recent, and we could not build a larger cohort. On top of this, the imbalance between long survivors and patients with a poor response did not let us build a predictive model at a time point other than 3 months. In clinical use, it would be more useful to predict a 6- or 12-month survival and the length of patient disease control. Since only 20% to 30% of the patients treated with PD-1/PD-L1 inhibitors will respond to treatment, the number of included patients must be larger to use machine-learning methods in this context. The number of patients was even lower when we used the genomic data since this genomic analysis is not conducted systematically in current practice. The combination of genomics with radiomics did increase the number of features embedded in the model but decreased the number of observations. It is quite interesting to show that the variety of data may improve the quality of the prediction model, but further work in larger cohorts is mandatory to confirm our results.

Second, a retrospective study such as ours implies highly varying imaging protocols. Statistical harmonization methods such as ComBat (35, 36) could be useful in this context to address potential batch effects linked to acquisition protocol heterogeneities. In order to perform a batch correction, other studies have to be done to select the batch effect criterion. On the other hand, the model might well learn acquisition protocol heterogeneity. When the bias/variance dilemma is well-balanced, the classifier learns a general law rather than dataset specificities. Another limitation of our study pertains to the manual segmentation of the lesion. Reproducibility and repeatability could not be tested in this study because tumors were segmented by a single radiologist. Indeed, the segmentation of grade III/IV patients' lesions is challenging because of their varying localization, shape, and margins (37). In the same way, the effect of segmentation could not be studied. Semiautomatic volumetric segmentation in the future may help to increase inter- and intraobserver reproducibility (38) and may be the key to the routine use of radiomic models.

Third, the fact that the population studied in this work displayed a large range of tumor sizes and tumor volumes is recognized as a bad prognostic factor itself. In this context, further studies including patients with a comparable tumor volume—so as to evaluate its potentially confounding effect—are mandatory.

In conclusion, this pilot study showed that it is possible to use pretreatment CT scan radiomics to train prediction models for the response of stage III/IV NSCLC to PD-1/PD-L1 inhibitors. The use of genomics to enrich radiomics may increase the performance of radiomic models. The correlation between radiomics and HOT/COLD status may explain the capacity of radiomics to predict clinical outcomes. However, multicentric data sharing will be required to increase the number of data and more carefully evaluate overfitting and batch effects linked to the use of data acquired from non-standardized acquisition protocols.

## Data Availability

The original contributions presented in the study are publicly available. The data relating to these can be found here: National Center for Biotechnology (NCBI) Gene Expression Omnibus (GEO), https://www.ncbi.nlm.nih.gov/geo/, GSE161537.
